# Engineering Globin Gene Expression

**DOI:** 10.1016/j.omtm.2018.12.004

**Published:** 2018-12-18

**Authors:** Rachael Davis, Aishwarya Gurumurthy, Mir A. Hossain, Eliot M. Gunn, Jörg Bungert

**Affiliations:** 1 Department of Biochemistry and Molecular Biology, College of Medicine, UF Health Cancer Center, Genetics Institute, Powell Gene Therapy Center, University of Florida, Gainesville, FL 32610, USA

**Keywords:** hemoglobin, globin, locus control region, gene therapy, gene editing, zinc finger, TALEN, CRISPR/Cas9, hematopoiesis

## Abstract

Hemoglobinopathies, including sickle cell disease and thalassemia, are among the most common inherited genetic diseases worldwide. Due to the relative ease of isolating and genetically modifying hematopoietic stem and progenitor cells, recent gene editing and gene therapy strategies have progressed to clinical trials with promising outcomes; however, challenges remain and necessitate the continued exploration of new gene engineering and cell transplantation protocols. Current gene engineering strategies aim at reactivating the expression of the fetal γ-globin genes in adult erythroid cells. The γ-globin proteins exhibit anti-sickling properties and can functionally replace adult β-globin. Here, we describe and compare the current genetic engineering procedures that may develop into safe and efficient therapies for hemoglobinopathies in the near future.

## Main Text

### Hemoglobin, Globin Gene Regulation, and Hemoglobinopathies

Hemoglobin is a hetero-tetramer composed of two α and two β chains, each containing a heme group that reversibly binds oxygen.^1^ The composition of hemoglobin changes during development, which facilitates transfer of oxygen from the mother’s blood to that of the embryo and fetus. Adult hemoglobin (HbA) contains two α and two β chains, whereas fetal hemoglobin (HbF) contains two α- and two γ-globin chains. The α- and β-type globin genes are under control of super-enhancers (SEs) that mediate extremely high levels of expression during erythroid differentiation.^2^, ^3^, ^4^ The β-globin SE is called locus control region (LCR) and consists of five DNase I hypersensitive sites (HSs) that come in close proximity to promoters during activation of the globin genes.^5^ Furthermore, the LCR engages in looping interactions with the genes to promote high-level expression.^6^, ^7^ LCR HS2 contains the strongest enhancer activity, whereas HS3 harbors a dominant chromatin opening activity.^8^, ^9^

Hemoglobinopathies are among the most common genetic disorders in the human population and include sickle cell disease (SCD) and β-thalassemia.^1^ SCD results from a single base pair change in the adult β-globin gene leading to the replacement of a glutamic acid by a valine at position 6 of the protein.^10^ This amino acid replacement creates a hydrophobic pocket in the deoxygenated state that allows hemoglobin molecules to stick together, forming long polymers that deform red blood cells into a sickle-like shape. Sickled erythrocytes are rigid and block small blood vessels, causing severe complications including pain, inflammation, and stroke.^10^ Thalassemia exists when there is absence or reduction of globin chain synthesis.^1^ Cooley’s anemia is the most severe form and is characterized by the absence of adult β-globin production. Thalassemia also results from reductions in globin chain synthesis due to specific mutations in the coding region of the genes or mutations and deletions of positive regulatory DNA elements. Symptomatic β-thalassemias are more common than α-thalassemias due to the fact that there are four copies of the adult α-globin gene.^1^ Excess α-globin in β-thalassemic cells precipitates and causes damage to the erythrocyte membrane, eventually resulting in hemolysis. Failure to produce mature red blood cells leads to anemia and bone marrow deformation.^1^

Hereditary persistence of HbF (HPFH) is a condition characterized by elevated levels of HbF in adult individuals.^11^ Increased HbF not only reduces the overall proportion of sickled cells but also inhibits polymerization of deoxygenated sickle hemoglobin, thereby reducing symptom burden. When the HbF is present in erythrocytes in a pan cellular distribution, polymerization can be completely eliminated with as little as 30% total HbF concentration.^12^ HPFH is often caused by deletions or mutations of negative DNA-regulatory elements that repress the γ-globin genes in adult erythroid cells. Genome-wide association studies identified additional mutations causing HPFH.^13^, ^14^, ^15^ For example, transcription factor BCL11A emerged as a major repressor of γ-globin expression after mutations in BCL11A-regulatory DNA elements were associated with HPFH.^13^, ^16^ BCL11A binds to the β-globin gene locus and suppresses γ-globin gene transcription.^17^, ^18^, ^19^ Expression of BCL11A is under control of KLF1, which also activates adult β-globin expression.^20^ Heterozygous mutations in KLF1 cause HPFH phenotypes in humans.^21^

### Development of New Therapies for Hemoglobinopathies

**Figure 1:**
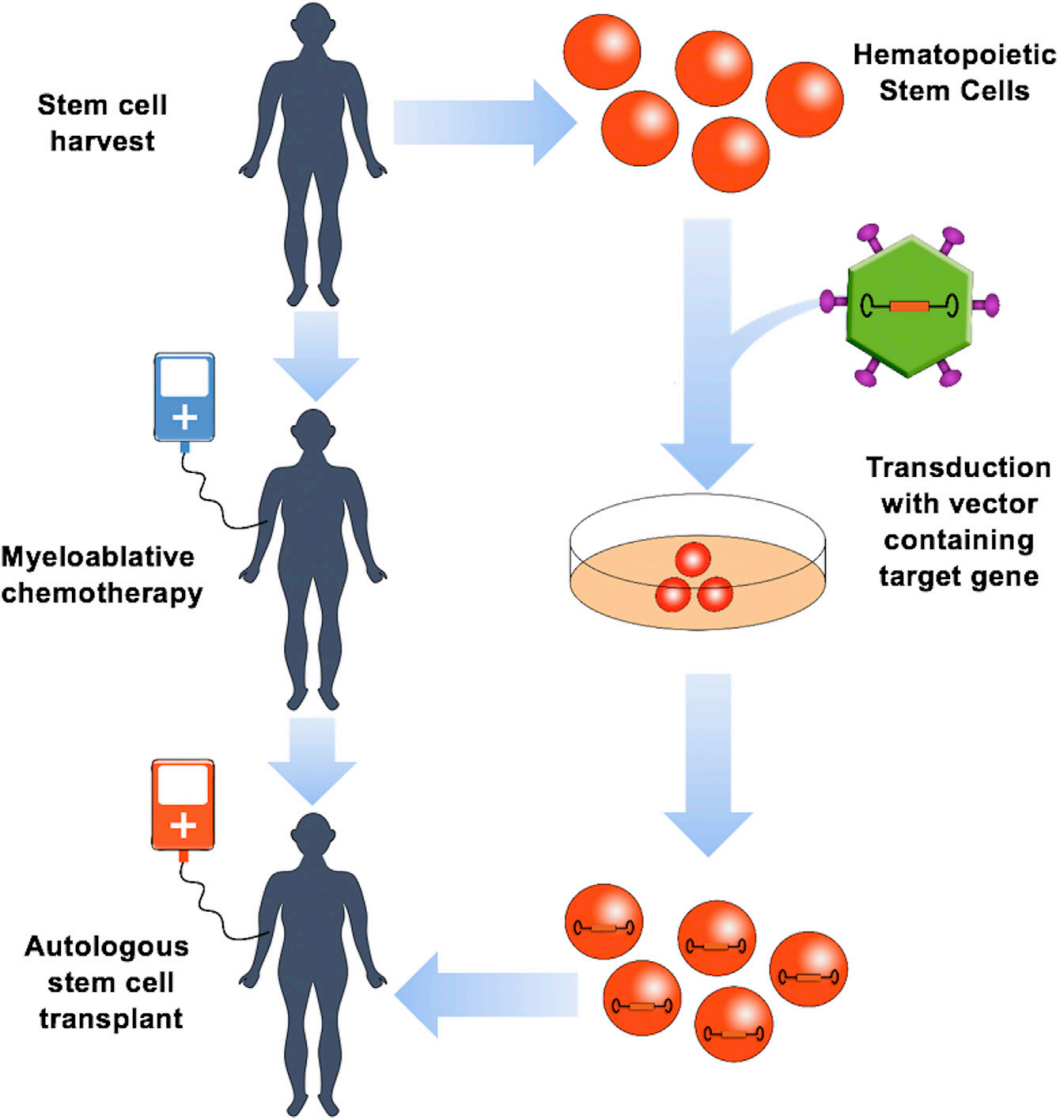
Human Hematopoietic Gene Therapy Hematopoietic gene therapy is unique in that progenitor stem cells can be removed from the patient, therapeutically manipulated, and then returned to the patient to establish long-lived self-perpetuating production of modified mature cells, thereby limiting exposure of off-target body systems to the modifying agents. Stem cells must first be mobilized with the aid of colony-stimulating factors and harvested from the patient typically via the use of apheresis. The cells are then modified *in vitro*. In this example, the cells are exposed to a viral vector capable of delivering a therapeutic gene. The patient must then receive myeloablative chemotherapy to provide physical space and reduced competition for the modified cells to engraft. The modified cells are then infused back to the patient, where they migrate to the bone marrow and engraft with the goal of establishing cell lines that will provide long-term, ongoing benefit to the patient.

Current treatments for hemoglobinopathies include hematopoietic stem cell (HCS) transplantation, blood transfusions, and hydroxyurea, which increases expression of HbF.^22^, ^23^ HSC transplantation provides a long-term cure but is restricted to major histochemistry complex (MHC)-matched donors and is associated with certain risks. Frequent blood transfusions lead to toxic iron overload and require chelation therapy. Only about 50% of sickle cell patients benefit from hydroxyurea.^22^, ^23^ These limitations highlight the need for the development of new therapies for hemoglobinopathies. There are many different experimental approaches, including the use of small molecules that enhance γ-globin expression, gene therapy, and genome editing.^22^, ^23^ Impressive progress has been made in all of these areas over the last decade. The use of small therapeutic molecules would be advantageous because of potential systemic delivery and low cost. Most of the small-molecule inhibitors target negative co-regulators involved in γ-globin gene silencing. For example, inhibitors of the lysine-specific histone demethylase 1 (Lsd1), a co-repressor recruited to the fetal γ-globin gene promoters, showed promising results in mouse and primate model systems.^24^, ^25^ A possible limitation with these small molecules is their potential to broadly interfere with the function of transcription co-regulators causing negative effects that may negate the therapeutic increase in HbF production. This review focuses on gene engineering approaches for the treatment of hemoglobinopathies, with a particular emphasis on gene therapy and genome editing. For both strategies, HSCs are harvested from the patient and transduced with a vector containing the therapeutic gene or transfected with genes or RNA of the gene editing system. The modified cells are subjected to quality control and then transplanted back to patients who have undergone myeloablative therapy (Figure 1).

### Globin Gene Therapy

The first attempt of human gene therapy for a hemoglobin disorder occurred in the early 1980s.^26^, ^27^ Almost 30 years later, this approach succeeded in a clinical trial in France.^28^ The field had to overcome several major obstacles including: (1) the development of efficient and safe procedures for the patient conditioning, as well as mobilization, manipulation, and transplantation of HSCs; (2) the development of safe and efficient delivery vehicles for therapeutic globin genes; and (3) the assembly of a combination of regulatory DNA elements that mediate high-level expression of the therapeutic genes without interfering with the expression of endogenous genes at the site of transgene integration.

In the past, several viral systems were explored for the development of globin gene therapy, including retrovirus, adenovirus, adeno-associated virus (AAV), and lentivirus.^29^, ^30^ Retroviral and adenoviral strategies proved to be inefficient or unsafe. Furthermore, AAV is too small to accommodate the therapeutic globin coding region and DNA-regulatory elements required to drive high-level expression. In addition, many AAV vectors are modified to prevent integration into the genome and are not suitable for HSCs. Lentivirus, on the other hand, efficiently transfects HSCs and allows incorporation of complex DNA-regulatory elements including LCR HSs, β-globin promoter, and 3′ enhancer, as well as insulator elements that shield the transgene from repressive chromatin and protect endogenous genes near the site of transgene integration from being activated by the transgene’s regulatory DNA elements.^29^, ^30^

Several potentially therapeutic genes have been targets of examination for gene therapies of β-thalassemia major and SCD including the wild-type adult β-globin gene, the fetal γ-globin gene, and mutant β-globin genes. The wild-type β-globin gene is used primarily for the treatment of β-thalassemia major. SCD benefits from the expression of a globin protein that exhibits efficient anti-sickling properties. This is an exploitable natural feature of fetal γ-globin and achievable with modified globin genes. Current gene therapy trials use either the γ-globin genes or a mutant adult β-globin gene that contains one (Q87) or three (AS3: D16, A22, and Q87) mutations.^31^, ^32^ Q87 and A22 disrupt lateral and axial contacts between hemoglobin tetramers providing the anti-sickling property, whereas D16 increases the affinity for α-globin conferring a competitive advantage over the sickle β-globin chain.

The therapeutic globin gene requires high levels of expression in both β-thalassemia major and SCD for clinical benefit. The design of vectors that confer high-level expression has been a tour de force over the years. This work included the identification and removal of DNA elements that negatively affected virus titer and the inclusion of insulator elements that protect the transgene from potentially negative effects at the site of vector insertion.^29^, ^30^ Insulators help to prevent the potential activation of oncogenes. The most commonly used insulator sequence, cHS4, originates from the chicken β-globin gene locus, where it functions as an enhancer blocker and a chromatin boundary.^33^, ^34^ It contains binding sites for the insulator protein CTCF and other proteins that prevent the spread of active or repressive chromatin marks. The cHS4 insulator has proven to be useful in gene therapy studies but revealed a negative effect on lentivirus titer and caused vector rearrangements that led to loss of insulators.^28^ Current research focuses on identifying insulator elements that function like cHS4 but do not affect production of the therapeutic virus particles.^35^

One of the most challenging aspects in the development of globin gene therapy vectors has been and continues to be the choice of regulatory DNA elements. The DNA sequence information driving expression of the therapeutic gene should be erythroid-specific and confer extremely high-level expression. The discovery of the β-globin LCR opened up new avenues to drive high-level expression of therapeutic genes; however, the LCR is more than 15 kb in size, making inclusion of the entire LCR difficult or impossible even in lentivirus vectors. Investigators instead include specific combinations of LCR HSs. Transgenic studies have shown that deletion of individual HSs can significantly impair the ability of the LCR to confer position-independent expression of the globin genes.^36^, ^37^ LCR HS2, HS3, and HS4 harbor strong enhancer activity, and a combination of LCR HS2 and HS3 demonstrated the ability to drive high-level expression in cell culture or in the context of transgenic mice.^38^, ^39^ Importantly, the Grosveld laboratory^9^ demonstrated that LCR HS3 harbors a dominant chromatin opening activity. Further studies demonstrated that an enhancer located downstream of the adult β-globin gene is important for high-level expression.^40^, ^41^ All of these studies informed the design of effective lentivirus vectors for the high-level expression of a therapeutic globin gene.

**Figure 2:**
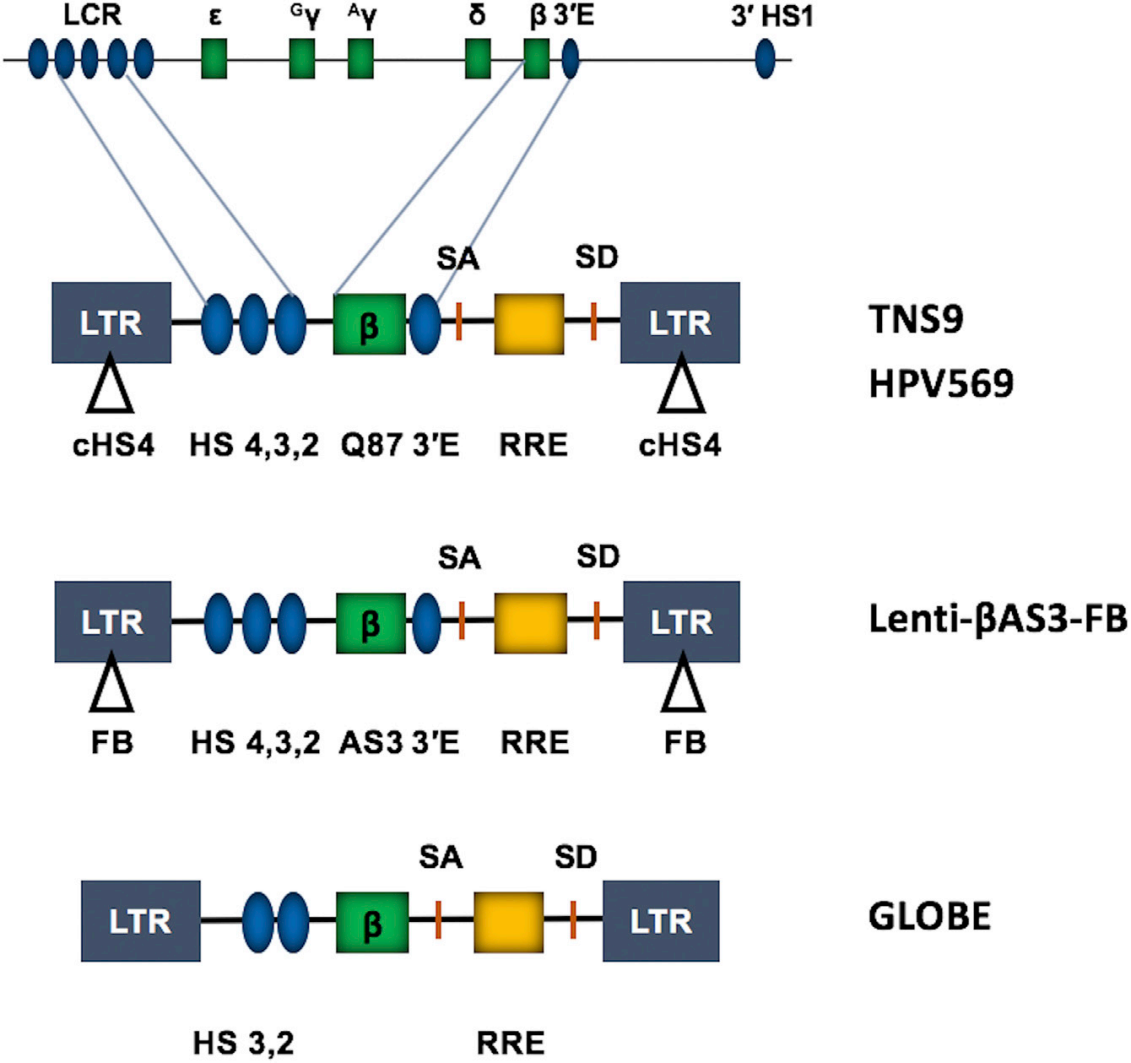
Lentivirus Vector Employed in Globin Gene Therapy The diagram on top depicts the human β-globin gene locus, which consists of five genes (green boxes) that are expressed in a developmental stage-specific manner. High-level expression of the adult β-globin gene is mediated by the LCR HSs and the β-globin 3′ enhancer (blue ovals). Functional elements within all lentivirus vector systems include the long-terminal repeats (LTRs; gray boxes), splicing acceptor (SA) and donor (SD) sites, and the rev-responsive element (RRE), a structured RNA required for efficient viral replication. The TNS9 vector contains large segments of LCR elements HS2, HS3, and HS4, a wild-type β-globin gene, and a β-globin 3′enhancer. The HPV569 vector is similar to the TNS9 vector but contains a mutant β-globin gene that encodes a protein with a T to Q substitution at position 87. In addition, this vector contains two copies of the chicken HS4 (cHS4) insulator sequence in the LTRs. The Lenti-βAS3-FB vector is similar to HPV569 but expresses a β-globin protein with three amino acid substitutions (AS3) and contains single FB insulators in the LTRs. The GLOBE vector contains the β-globin gene and two large segments of the LCR (HS2 and HS3).

The first proof-of-concept study demonstrated high-level expression of a globin gene driven by large fragments of LCR HS2, HS3, and HS4, plus the β-globin promoter and 3′ enhancer (TNS9; Figure 2) in a β-thalassemic mouse model.^42^, ^43^ A similar construct (HPV569) expressing the anti-sickling Q87 globin and also including two copies of the cHS4 inserted into the LTR U3 region was successfully used in the first human gene therapy trial.^28^ In this study (LG001), a myeloid cell clone in which the virus integrated into the *HMGA2* gene locus and activated a truncated HMGA2 protein became the dominant clone over a period of 2 years. This was fortuitous, but the restriction of high HMGA2 expression to the erythroid lineage also demonstrates that the strong LCR elements potentially activate genes at the site of virus integration.

Subsequent globin gene therapy experiments did not produce evidence for clonal dominance but instead revealed heterogeneous integration patterns that remained diverse over time.^29^, ^30^, ^44^ The follow-up study to LG001 used a similar lentiviral vector, BB305, which differed by removal of cHS4 insulators and the addition of the cytomegalovirus promotor. These changes promoted higher vector titers and a significant increase in unique integration sites. This approach led to the complete resolution of transfusion dependence in compound heterozygous β-thalassemia patients.^45^ Patients with homozygous β^0^/β^0^ had significant reduction in transfusion requirements, but expression of transduced β-globin did not consistently reach levels necessary for complete transfusion independence.^45^ This vector has also been used with some success in patients with SCD, resulting in complete resolution of clinically significant sickle cell-related disease manifestations.^46^

There are many ongoing globin gene therapy trials each using slightly different lentivirus constructs (Figure 2). For example, the Lenti-βAS3-FB vector consists of the AS3 β-globin gene and FII-BEAD-A (FB) insulators.^47^ The 77-bp FB insulator contains the enhancer-blocking elements of cHS4 and additional sequences derived from the human T cell receptor α/δ BEAD-1 insulator.^48^ Studies have shown that this new insulator element reduced the transforming potential of retroviral and lentiviral vectors. The GLOBE vector only contains LCR elements HS2 and HS3, and lacks the β-globin 3′enhancer as well as insulator elements.^49^, ^50^ It has been successfully used in correcting β-thalassemia major in cells from pediatric patients. The lack of insulator in the GLOBE vector may be a concern with respect to the activation of genes near the vector integration site; however, so far, there is no evidence that this vector harbors transforming potential.

### Globin Gene Editing

**Figure 3:**
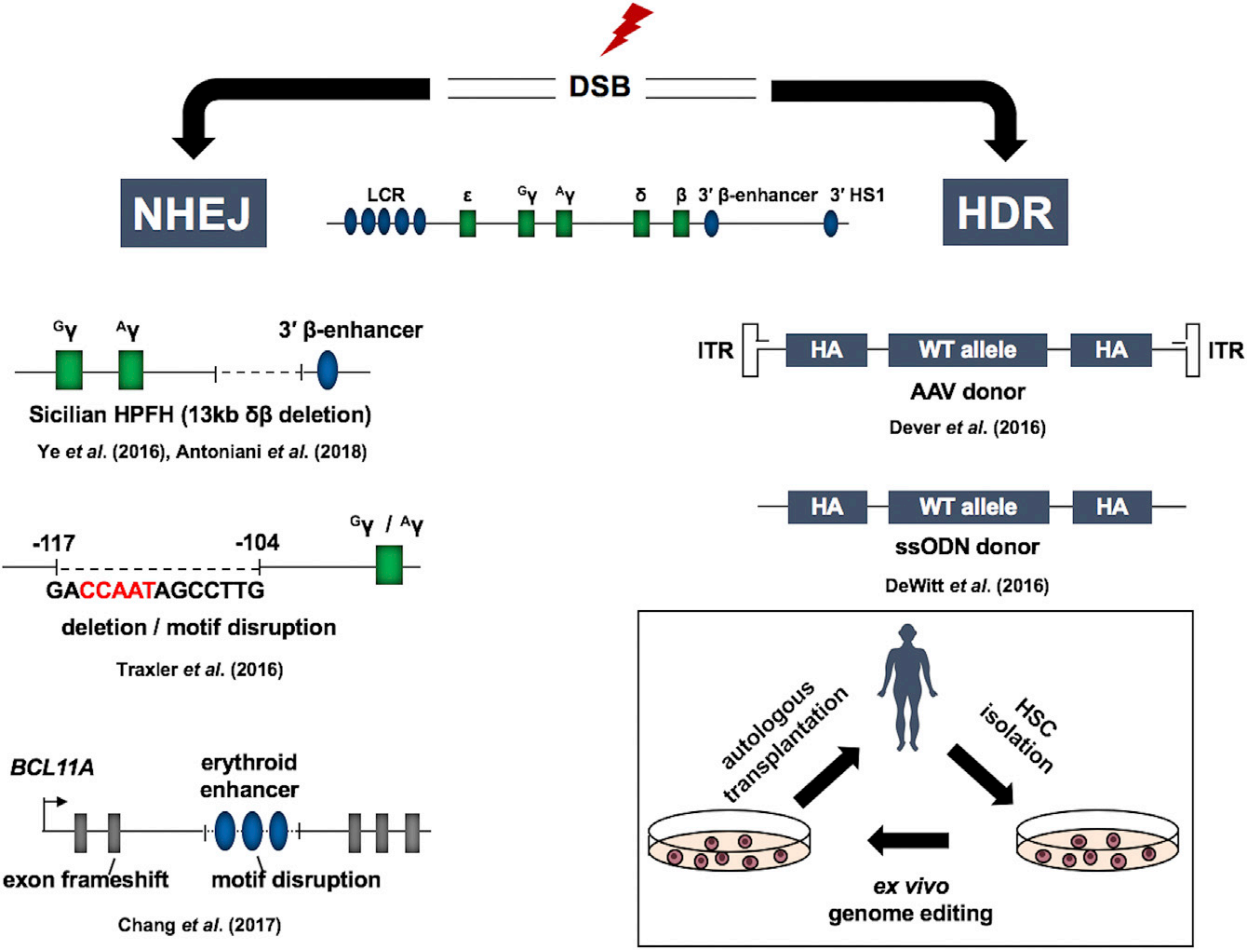
Elevation of γ-Globin Expression by Genome Editing Nucleases targeted to genomic sites create double-strand breaks (DSBs) that can be repaired by non-homologous end joining (NHEJ) or by homology-directed repair (HDR). Shown on the left are three examples for deletions causing increases in fetal hemoglobin production. The first shows the creation of a 13-kb deletion encompassing the δ- and β-globin gene. This deletion removes negative regulatory elements and positions the β-globin 3′ enhancer in close proximity to the γ-globin genes. The second example shows deletion of a repressor binding site in the γ-globin gene promoters. The third example shows creation of a frameshift in the coding region or a deletion of a positive DNA-regulatory element in one of the BLC11A erythroid-specific enhancers. Shown on the right are two examples for HDR-mediated correction of the sickle cell mutation. The first example shows an adeno-associated vector (AAV) that delivers the homologous arms (HAs), the therapeutic donor DNA, and a selectable marker (ITR: inverted terminal repeats). The second example shows a single stranded oligodeoxynucleotide containing 5′ and 3′ homologous arms (HAs) and the wild-type β-globin sequence. The diagram on the bottom right outlines the major steps involved in genome editing using autologous cell transplantation.

Genome editing represents an alternative strategy for the treatment of hemoglobinopathies.^51^ It is based on the sequence-specific targeting of a nuclease to the genome and the repair of the double-strand break (DSB) by either non-homologous end joining (NHEJ) or homology-directed repair (HDR) (Figure 3). NHEJ alters the genome by small insertions or deletions and is induced by targeted DSBs to remove DNA-regulatory elements or to prevent expression of a protein. Therapeutic double-stranded or single-stranded DNA is provided together with a targeting nuclease to mediate HDR in which a mutant sequence can be replaced by the wild-type sequence. The targeting molecules can either be synthetic DNA-binding proteins, e.g., zinc finger (ZF) and transcription activator-like effector (TALE) proteins, or RNA in the context of the CRISPR/Cas9 system.^52^ ZFs or TALE proteins are usually fused to the Fok1 nuclease generating ZF-nuclease (ZFN) or TALE-nuclease (TALEN).^52^ One ZF interacts with 3 bp in the DNA, and 3 or 4 ZF-containing proteins are generated to target a 9- or 12-bp target sequence. The proteins are fused to a portion of Fok1. Two 3- to 4-ZF proteins are then used to reconstitute Fok1 activity at a specific target sequence 18–24 bp long. The reconstitution of Fok1 nuclease activity at the target site by two ZF proteins reduces off-site nuclease activity. TALENs bind to 15 bp DNA; the combined target sequence of two TALENs that reconstitute Fok1 would be 30 bp. In the CRISPR/Cas9 system, the guide RNA targets the Cas9 nuclease to an 18-bp target sequence,^53^ a sequence long enough to represent a unique signature in the human genome. Among the three systems, CRISPR/Cas9 has advantages with respect to ease of design and expenses. All three systems have limitations including the possibility of generating off-target DSBs and chromosomal rearrangements.^52^, ^54^ Current efforts focus on generating nucleases with increased specificity. Genome editing represents a great promise for the development of new and globally applicable therapies for hemoglobinopathies.

The ideal cure for a genetic disorder would be to correct the mutation *in vivo* without leaving any trace of the tools used in the process. Targeted nuclease-mediated HDR would thus be the perfect strategy to combat SCD and many of the thalassemias. In proof-of-concept studies, several laboratories demonstrated efficient HDR-mediated repair of the sickle cell mutation in human CD34^+^ or other hematopoietic stem and progenitor cells (HSPCs).^55^, ^56^, ^57^ Dever et al.^55^ used CRISPR/Cas9 to induce a DSB near the sickle cell mutation and an AAV vector to deliver a homologous DNA fragment that not only contained the wild-type β-globin gene sequence but also a selectable marker gene (Figure 3). This strategy allowed for the enrichment of repaired cells by more than 90%. In contrast, DeWitt et al.^56^ and Hoban et al.^57^ demonstrated efficient HDR of the sickle cell mutation using a selection-free approach. The intrinsic problem with HDR-mediated therapy is that NHEJ is more frequently used to repair a DSB compared with HDR. In the absence of selection, most cells will carry insertions or deletions after repair of the DSB, which could result in a β^0^ thalassemia allele. This may even occur in the replaced template if the targeting is not specific for the endogenous DNA and if the targeted nuclease is still present after HDR takes place. For this reason, investigators explore the possibility of using targeted nucleases in conjunction with NHEJ to inactivate proteins or DNA-regulatory elements implicated in the repression of γ-globin gene expression.

There are many different mutations associated with HPFH including point mutations that disrupt repressor binding at the γ-globin promoter, deletions of relatively large regions in the β-globin gene locus that bring enhancer elements close to the fetal γ-globin genes, and mutations outside of the globin gene loci that affect the function of *trans*-acting repressor proteins of γ-globin expression^11^. Two groups generated a 13-kb deletion recapitulating the Sicilian HPFH mutation in CD34^+^ cells using CRISPR/Cas9 (Figure 3).^58^, ^59^ This deletion juxtaposes the β-globin 3′enhancer close to the fetal γ-globin genes and also removes negative-acting elements located upstream of the δ-globin gene.^60^, ^61^ Ye et al.^58^ found that erythroid colonies differentiating from cells with the 13-kb deletion expressed elevated levels of γ-globin. No evidence of off-targeting was observed. Antoniani et al.^59^ found that introducing the deletion into HSPCs from sickle cell patients elevated HbF synthesis and reduced the sickling phenotype in erythroblasts.

Traxler et al.^62^ deleted a 13-bp DNA sequence located about 100 bp upstream of the γ-globin promoter in CD34^+^ HSPCs from three patients with SCD (Figure 3). This region has been shown to interact with transcription repressors including proteins that bind to the CCAAT-box, the DRED complex, which binds to a direct repeat element via nuclear receptor proteins TR1 and TR2, and BCL11A.^18^, ^19^, ^63^ DRED contains co-repressors including LSD1, a histone demethylase specific for H3K4.^64^ Importantly, deletion of the repressor binding site led to reversal of the sickle phenotype and an increase in the number of F cells.^62^ Li et al.^65^ used CRISPR/Cas9 to delete a similar sequence in the context of HSPCs derived from human β-globin YAC transgenic mice. The cells were transplanted back into irradiated mice, and high levels of γ-globin expression were observed even after a secondary transplant.

Several investigators have explored BCL11A as a target for therapeutic intervention of hemoglobinopathies using genome editing approaches. BCL11A is required for the function of several cell types, and ablation of BCL11A function can cause undesirable downstream effects, such as leukemia and lymphoma; therefore, targeting BCL11A should be restricted to the erythroid lineage.^66^ Several erythroid-specific enhancer elements are present in the *BCL11A* gene locus.^67^, ^68^ Chang et al.^68^ used ZFNs to disrupt the BCL11A coding sequence or to delete an erythroid-specific *cis*-regulatory DNA element, a GATA motif, in one of the *BCL11A*-associated erythroid enhancers (Figure 3). Disruption of the coding region reduced the differentiation potential of erythroid cells; however, disruption of the GATA motif decreased expression of BCL11A and led to elevated HbF production without impairing erythropoiesis. The same strategy was successful in HSPCs from thalassemic patients.^69^

The current gene therapy trials and studies using genome editing are promising, and it seems feasible that some of the pursued strategies will reliably cure hemoglobinopathies in the future; however, problems associated with mobilization of HSPCs, especially from thalassemic patients, with potential off-targets during genome editing and with changes of gene expression at the insertion site of globin gene therapy vectors remain to be addressed. In addition, targeted nucleases have been shown to create unwanted chromosomal rearrangements. This may be due to the presence of sequences of partial homology, as was shown for rearrangements between the δ- and β-globin genes after induction of DSBs at the β-globin gene.^70^

### Alternative Strategies

**Figure 4:**
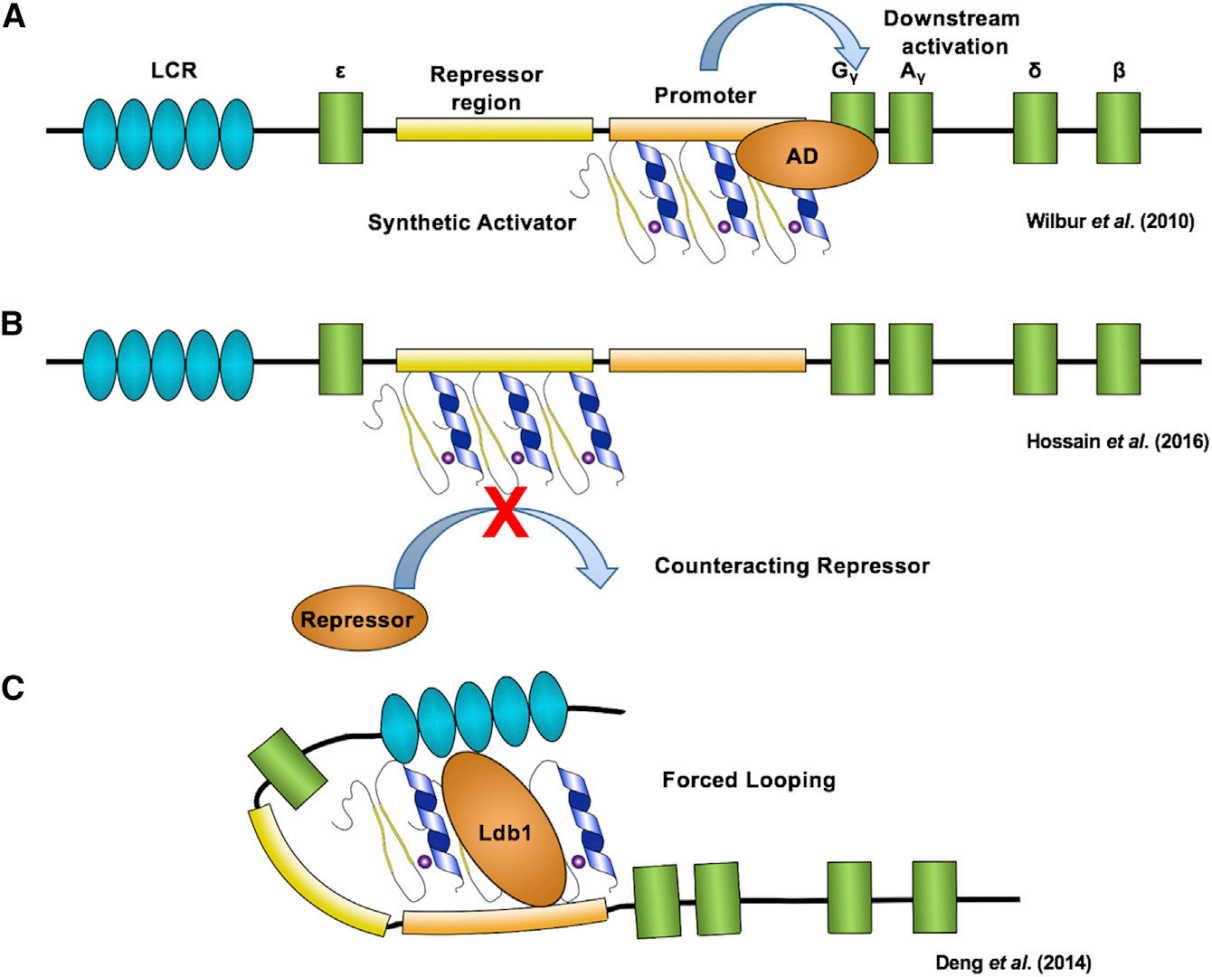
Alternative Approaches Using Synthetic Zinc Fingers (A) Synthetic ZF activator binding to promoter. ZFs fused to an activation domain (AD) such as VP64 and specific for the γ-globin genes brings the activation domain into close proximity of the promoter, resulting in activation of γ-globin transcription. (B) Decoy ZF protein binding to a repressor region. ZFs can be designed to bind target sites for known repressors. The ZF protein provides competitive inhibition of the repressor elements, thereby activating fetal hemoglobin. (C) ZF protein facilitated chromatin looping. ZF protein fused to the dimerization domain of Ldb1 and specific for the γ-globin gene promoter can mediate chromatin looping, which brings the LCR into proximity to the promoter allowing for activation of fetal hemoglobin.

There are also a number of unconventional strategies that are being explored for the development of new therapies for hemoglobinopathies (Figure 4). Wilber et al.^71^ fused the strong VP64 activation domain to a ZF protein targeted to the γ-globin gene promoters. VP64 is a tetrameric repeat of the minimal activation domain of herpes simplex virus (HSV) viral protein (VP) 16.^72^ Lentivirus-mediated delivery of the artificial activator enhanced expression of HbF up to 20% of total hemoglobin.^71^

High-level globin gene expression is mediated by the LCR, which comes in close proximity to the genes, suggesting a looping mechanism for globin gene activation.^6^ One of the proteins involved in mediating interactions between the LCR and the globin genes is Ldb1, a co-factor recruited by a GATA1/Tal1 protein complex.^73^ Ldb1 has a self-associating dimerization domain.^74^ Fusion of the Ldb1 self-associating domain to a synthetic ZF protein targeted to the γ-globin promoter activated γ-globin expression in adult erythroid cells; thus, forced looping can redirect LCR-mediated transcription activation from the adult to the fetal globin genes.^75^

ZF proteins have been used without effector domains to block the binding of a repressor complex to the γ-globin gene promoter.^76^ A DNA sequence located about 567 bp upstream of the Gγ-globin gene has been associated with HPFH and was shown to recruit a GATA1-containing co-repressor complex.^77^, ^78^ Targeting a ZF-DNA-binding domain (ZF-DBD) to this site elevated γ-globin expression in peripheral blood mononuclear cells (PBMCs) derived from normal and sickle cell patients.^79^ ZF-DBDs can be directly delivered to erythroid cells and localize to the nucleus even in the absence of a nuclear localization domain.^80^, ^81^

Thalassemia major is associated with an imbalance of globin chain production. The excess α-globin proteins precipitate out and damage the differentiating red blood cells. Mettananda et al.^82^ used CRISPR/Cas9 to delete one of the α-globin enhancers (MCS-R2). Differentiating β-thalassemic CD34^+^ HSPCs carrying a heterozygous deletion of MCS-R2 exhibited α/β-globin ratios similar to wild-type cells and lower than the β-thalassemic cells. Using xenografts, the authors demonstrated that the edited cells were long-term repopulating HSCs.

Investigators were recently able to show that triplex-forming peptide nucleic acids (PNAs), which interact with DNA via Watson-Crick and Hoogsteen base pairing, can induce HDR at the β-globin gene in CD117^+^ HSPCs.^83^ Stem cell factor, the c-Kit ligand, stimulated HDR in the cells by upregulating expression of DNA repair genes. Injection of nanoparticles containing the PNAs and therapeutic donor DNA plus stem cell factor (SCF) into thalassemic mice reduced the disease phenotype and resulted in 7% correction of the β-globin gene in HSCs. The authors further demonstrated that *in utero* delivery of the PNA and donor DNA via nanoparticles led to post-natal elevated β-globin gene expression.^84^ This strategy represents a promising minimally invasive and virus-free approach.

Variant CRISPR/Cas9 systems are used for site-specific base editing.^85^ This system uses a nuclease-defective Cas9 protein that is expressed as a fusion with a transaminase. A common β-thalassemia mutation in China and Southeast Asia is HbB-28 (A–G), which changes the TATA box and dramatically reduces β-globin transcription.^86^ Liang et al.^87^ targeted the Cas9-transaminase to the HbB-28 region in cells from thalassemic patients and observed frequent G-to-A conversions.^86^

### Conclusion

Current advances in the development of effective gene and genome editing therapies for hemoglobinopathies are impressive and will likely lead to clinical benefits in the near future. However, whether these therapies will be applicable to the large number of individuals afflicted by hemoglobinopathies is uncertain. In addition to the gene engineering approaches described here, efforts are under way to develop small drugs that reactivate γ-globin expression. Promising results have been obtained in mice and primates using an inhibitor of LSD1.^25^ Furthermore, recent studies implicated the heme-regulated eIF2α (HRI) in the repression of γ-globin gene expression.^88^ Thus, HRI inhibitors may represent another class of drugs that elevate γ-globin expression.
